# A New Era of Epidemiology: Digital Epidemiology for Investigating the COVID-19 Outbreak in China

**DOI:** 10.2196/21685

**Published:** 2020-09-17

**Authors:** Zonglin He, Casper J P Zhang, Jian Huang, Jingyan Zhai, Shuang Zhou, Joyce Wai-Ting Chiu, Jie Sheng, Winghei Tsang, Babatunde O Akinwunmi, Wai-Kit Ming

**Affiliations:** 1 Department of Public Health and Preventive Medicine School of Medicine Jinan University Guangzhou China; 2 Faculty of Medicine International School Jinan University Guangzhou China; 3 School of Public Health LKS Faculty of Medicine The University of Hong Kong Hong Kong China; 4 MRC Centre for Environment and Health Department of Epidemiology and Biostatistics, School of Public Health, St Mary’s Campus Imperial College London London United Kingdom; 5 College of Economics Jinan University Guangzhou China; 6 Center for Genomic Medicine Massachusetts General Hospital Harvard University Boston, MA United States; 7 Pulmonary & Critical Care Medicine Unit, Asthma Research Center Brigham and Women’s Hospital Harvard Medical School Boston, MA United States

**Keywords:** digital epidemiology, COVID-19, risk, control, public health, epidemiology, China, outbreak, case study

## Abstract

A novel pneumonia-like coronavirus disease (COVID-19) caused by a novel coronavirus named SARS-CoV-2 has swept across China and the world. Public health measures that were effective in previous infection outbreaks (eg, wearing a face mask, quarantining) were implemented in this outbreak. Available multidimensional social network data that take advantage of the recent rapid development of information and communication technologies allow for an exploration of disease spread and control via a modernized epidemiological approach. By using spatiotemporal data and real-time information, we can provide more accurate estimates of disease spread patterns related to human activities and enable more efficient responses to the outbreak. Two real cases during the COVID-19 outbreak demonstrated the application of emerging technologies and digital data in monitoring human movements related to disease spread. Although the ethical issues related to using digital epidemiology are still under debate, the cases reported in this article may enable the identification of more effective public health measures, as well as future applications of such digitally directed epidemiological approaches in controlling infectious disease outbreaks, which offer an alternative and modern outlook on addressing the long-standing challenges in population health.

## Introduction

A pneumonia-like coronavirus disease (COVID-19) outbreak caused by a newly identified coronavirus, SARS-CoV-2, swept across China in early 2020. As of early June, 215 countries or regions have reported confirmed cases, with 6,799,713 confirmed cases and 397,388 deaths, and a case fatality rate over 5.84% worldwide [1]. With the increasing incidence of confirmed cases, corresponding spread control policies and emergency actions are taking place. Holiday travel related to the Spring Festival in China has led to great difficulties in tracking suspected cases for outbreak control.

Conventional epidemiology dating back to the 1800s mainly relies on health-related data such as information gathered within health care systems, medical records, or insurance systems. Such data can only be collected and recorded from diagnosed or treated patients; therefore, it would be outdated and hinder the corresponding management efforts upon the abrupt outbreak of infectious diseases [2].

The public health measures that showed effectiveness in previous infection outbreaks (ie, mass use of face masks, social distancing, and home quarantine) were also implemented in the COVID-19 outbreak. Although the effectiveness of these public health measures in this outbreak is not clear, the availability of multidimensional media network data can provide an alternative outlook that takes advantage of the recent rapid development of information and communication technologies, allowing for better tracing and control of the disease spread. The quantity and dimensionality of data have substantially increased along with the continued development of technologies (eg, telecommunication), revolutionizing the way we communicate. Such technologies have shown great potential in terms of convenience and precision for the surveillance and modelling of infectious diseases such as influenza and severe acute respiratory syndrome, through extracting information from electronic health (eHealth), electronic payments, the internet, and social media [3,4]. This also brings epidemiology into a new era, that of so-called digital epidemiology [5], where digital data or data that were generated outside of the public health system are used, as proposed by some scholars [6]. Social media provides much of the data generated on the internet; by examining the search index or the texts posted, researchers can foresee the outbreak of an infectious disease. If certain keywords were searched for many times during a short period of time, this could indicate an infectious disease in the community; Google Flu Trends (Google Inc) makes use of this type of data [7-9]. Moreover, the spatiotemporal data related to individual behaviors can be extrapolated from the use of electronic payments, cellular service, or social media to study the distribution, incidence, and etiology of a disease, contributing to disease prediction and prevention [7,10-12]. However, some scholars in digital epidemiology have excessively used the internet, web-based systems, or network surveillance of media information, which may be biased and constrained by information overload, false reports, a lack of specificity of signals, and sensitivity to external forces [10].

**Figure 1:**
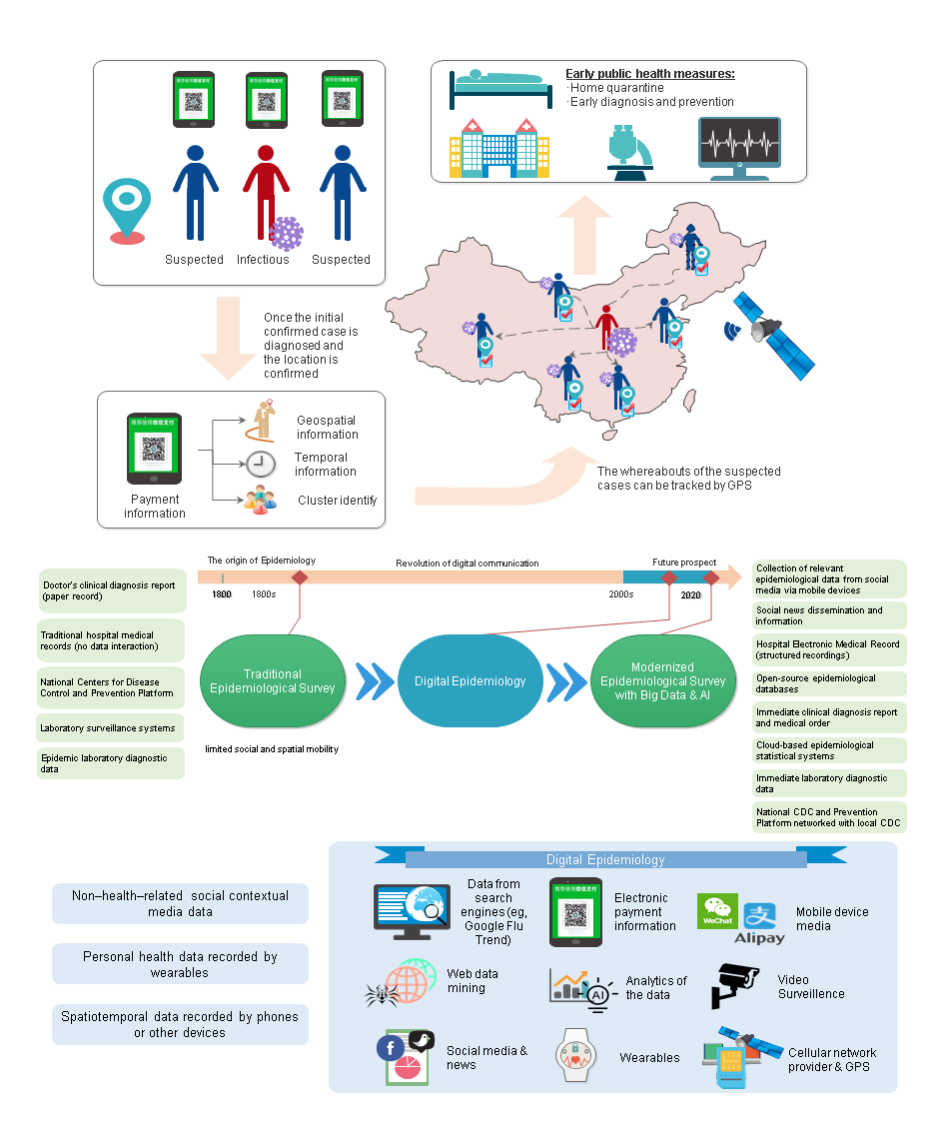
An infographic illustrating the development of digital epidemiology and its application in controlling infectious disease epidemics. CDC: Centers for Disease Control.

**Figure 2:**
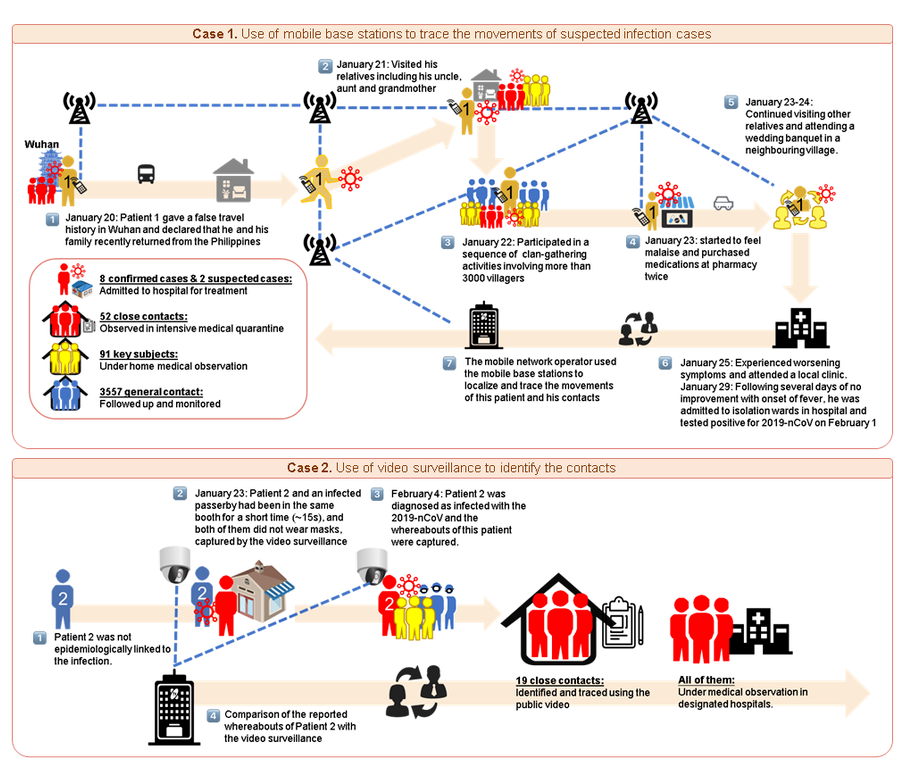
The application of digital epidemiology in the outbreak of COVID-19. Case 1: Use of mobile base stations to trace the movements of suspected infection cases. Case 2: Use of video surveillance to identify the contacts.

Nowadays, advances in mobile applications have enabled users to perform daily activities on their mobile phones, including making electronic payments and checking social media. The data on each activity performed, including the location of the mobile user, were also stored (Figure 1). Generally, there are three types of electronic data streams in the field of epidemiology, namely medical encounter data (eg, electronic records of medical institutions), participatory syndrome data (eg, personal health data, data from the population), and nonhealth digital data (eg, data from internet search engines, social media, or mobile use) [13]. The everyday movements of individuals create a dynamic link that connects people, which can be used to study the geographical spread and sustained transmission of infectious diseases [5]. In the past, population movements were traditionally estimated using travel surveys, road networks, or small-scale GPS studies, which have long hindered efforts to understand these dynamics [5]. Diverse types of digital trace data may enhance exposure measurement and facilitate strong tests of specific routes of transmission [5]. These data sources, if used appropriately, can provide preliminary and timely information about disease outbreaks and related events around the world. Furthermore, these sources enable a reduced time between initial detection of an outbreak and formal recognition of an outbreak, thus allowing for a more expedited response to such public health threats [14]. Since the epidemic spread is related to location-specific human contact patterns [15,16], it is deemed that more accurate estimates of transmission routes and the number of infection cases can be achieved by using available big data derived from mobile phones and video surveillance. Here, we present two publicly reported cases of COVID-19 in China that demonstrated the significant role that digital data can have in modernizing epidemiological investigation, showing the potential of guiding public health measures accordingly (Figure 2).

By using a phone carrier’s mobile phone tracking system and scrutinizing the data transmission between different base stations under the authorization of the local government, 3557 people were identified as general contacts and 8 people were confirmed as having infections. Strict measures were then undertaken: 8 confirmed cases and 2 suspected cases were admitted to hospital to receive treatments, 52 close contacts were observed in intensive medical quarantine, 91 key subjects received home medical observation, and all 3557 general contacts were followed up and monitored.

## Case 1

A male, a resident of Village A, City A, China, was diagnosed with COVID-19 on February 1, 2020 [17-19], after returning from Wuhan, where he ordinarily lives and works. To avoid unnecessary interruption to his schedule, he claimed that he and his family recently returned from the Philippines rather than Wuhan when they arrived at the village on January 20, without symptoms, prior to the lockdown of Wuhan (Figure 2). During the following days, he resided with his father and younger brother in the village and was involved in several activities. On January 21 and 22, he visited his relatives and attended a series of clan-gathering activities that more than 3000 people partook in. Starting on January 23, he felt malaise. He purchased medications at a pharmacy twice on January 23. Despite his symptoms, he continued visiting other relatives on the same day. On January 24, he attended a wedding banquet in a neighboring village. He experienced worsening symptoms on January 25 and decided to attend a local clinic. It was recommended that he undergo a home quarantine given his lack of fever. Following several days of no improvement and the onset of fever, he was admitted to an isolation ward in a hospital on January 29 and tested positive for COVID-19 on February 1.

## Case 2

A 56 year old male, living in Town B, City B, China, was diagnosed as positive for COVID-19 on February 4, 2020, and was quarantined and received treatment in a designated medical institution. Through traditional epidemiological investigation methods, this patient was determined to have no history of residence or travel in the epidemic area and no exposure to wild animals in the 14 days before the onset of symptoms. In addition, he had no acquaintances with confirmed cases in his local district. However, the activities of this patient were captured by video surveillance. After referring to the videos, it was determined that the patient spent a short period standing near a stranger at the same booth in a farmer’s market at 7:47 AM on January 23. They were not wearing face masks. This stranger was in fact a confirmed case living in the same district.

Using video surveillance, the whereabouts of this patient were retrieved, which resulted in the identification of 19 subjects with close contact, who were then put under observation in designated hospitals to prevent further contamination.

## Discussion

These two cases are examples of the successful application of emerging technology in monitoring people’s movements during disease outbreaks, with the potential to offer near real-time estimation of disease-related activities and fast identification of potentially infected subjects. The surveillance work in both cases was led by the local governments, and the privacy of the subjects remained protected and personal information was not leaked; the information was only accessible by designated authorities within the local governments.

During the COVID-19 outbreak, there has been general agreement regarding the lack of readiness for such a viral outbreak. Although China’s government introduced strict measures to restrict gathering and travel during the outbreak, the virus still spread due to its high infection rate, even during the incubation period. The outbreak could have been better controlled if better surveillance systems and high-end technologies were used to incorporate spatiotemporal movement data in models of the potential transmission patterns. The outbreak of COVID-19 has prompted a discussion on the incorporation of digital data in epidemiological research. The use of digital data can enhance traditional epidemic surveillance as well as digital epidemiology–directed applications, including incident infections, viral sequencing, improved infectious disease outbreak predictions, suspected contacts detection, early prevention and management, real-time numerical forecasting of pandemics, and evaluating the effectiveness of disease response strategies or interventions [13,20-24]. The use of spatiotemporal information generated by the daily usage of online communication tools, such as WeChat and Alipay, could play an important role in controlling the spread of this disease and others, if properly used. In China, a color‐coded health code and travel card system was created. The system tracks where citizens have been during the last 14 days through phone carriers, whose system logs can determine whether a given citizen’s phone connected to base stations in high-risk areas. Thus, the system will note which citizens have been to high-risk regions, and the provided code then dictates where citizens can go (ie, whether they should continue quarantining or are able to leave the house) [25,26].

With the rapid development of China's economy and the widespread adoption of cell phones, mobile payment systems have also developed rapidly. There are two mobile payment operators, Alipay and WeChat, which currently cover more than 90% of the domestic market in China, and they are leaders in the field of third-party payment. WeChat and Alipay are secure and convenient, and they have penetrated every aspect of people’s lives (eg, transactions, online shopping, self-service, public transport, and personal finances) [27]. These payment systems also obtain multidimensional data from users, including payment information, GPS information, and social media information [27], which can be used to help monitor and control the spread of infectious diseases.

Moreover, the popularization of wearable devices has enhanced our ability to collect data regarding spatial and temporal aspects of human movements with higher precision [28], affording a much more detailed identification and stratification of social behaviors [29], complementing previous work based on large-scale surveys and self-reported information [5,30]. These data provide one of today’s most exciting opportunities to study human mobility and its influence on disease dynamics [31].

Despite the merits of using such technologies and data, several concerns still remain. First, validation of real-world data should be considered because the extraction of meaningful data from social networks has always been challenging [13,22,32]. Second, although the cases discussed in this article used a novel stream of data, the investigation methods and strategies were still outmoded. Therefore, how such digital data can be more effectively used and analyzed, using analytic algorithms with scientific justification and statistical power, requires further exploration [33]. Third, the legal and ethical aspects of using digital data remain questionable. The use of digital data has been extensively debated worldwide. Some of the electronic traces that we leave behind as digital citizens are meant to be public, while others are not, resulting in ethical and legal challenges [34,35]. Regarding the ethics surrounding public health and digital epidemiology, there are the competing issues of protecting and promoting the health of populations and potentially causing individual harm as a result of collecting data from digital networks [35,36]. These two COVID-19 cases in China serve as a successful example of how digital data generated by companies and used by local governments can be used to mitigate the spread of COVID-19, by identifying people who have travelled to high-risk areas or tracing people who have contacted people with COVID-19. Indeed, such data should be covered by data-protection regulations, and privacy and confidentiality should be guaranteed, but there would have been no other way for the relevant authorities to obtain this data. In addition, the issue of privacy has been extensively discussed [37-41]. Fourth, false discrimination has been demonstrated in previous studies as a result of incorrect identification of internet users; thus, an improvement in this aspect is required. Fifth, multidimensional data such as the data extracted from electronic payments in China may not be available in other countries; thus, further exploration of local contexts is needed. Finally, issues related to data access, data sharing, user privacy, and data security still require attention, yet public health takes precedence in such situations. The two abovementioned cases serve as perfect examples of local governing bodies taking part in epidemiological research using digital data. Therefore, we hold an optimistic view on the further implementation of digital epidemiology for disease outbreaks, especially following related achievements and experiences during the COVID-19 outbreak.

This article demonstrated the plausibility of using digital epidemiology to control and prevent infections, based on two real-life cases during the COVID-19 outbreak in China. Taking advantage of emerging information and communication technologies and accessible multidimensional spatiotemporal data for monitoring people’s movements, this modernized epidemiological approach can help shed more light on the pattern of disease spread and contribute to identifying more effective public health measures to mitigate the negative impact of COVID-19. It can also be used to identify long-standing challenges in population health.

## Abbreviations

COVID-19: coronavirus disease
